# Learning to Rapidly Re-Contact the Lost Plume in Chemical Plume Tracing

**DOI:** 10.3390/s150407512

**Published:** 2015-03-27

**Authors:** Meng-Li Cao, Qing-Hao Meng, Jia-Ying Wang, Bing Luo, Ya-Qi Jing, Shu-Gen Ma

**Affiliations:** 1 Institute of Robotics and Autonomous Systems, Tianjin Key Laboratory of Process Measurement and Control, School of Electrical Engineering and Automation, Tianjin University, Tianjin 300072, China; E-Mails: menglicao@tju.edu.cn (M.-L.C.); wjy0709@tju.edu.cn (J.-Y.W.); roice@tju.edu.cn (B.L.); jingyaqi@tju.edu.cn (Y.-Q.J.); shugen@se.ritsumei.ac.jp (S.-G.M.); 2 Department of Robotics, Ritsumeikan University, 1-1-1 Nojihigashi, Kusatsu-Shi 525-8577, Japan

**Keywords:** chemical plume tracing, reinforcement learning, collaborative learning, behavior-based robotics

## Abstract

Maintaining contact between the robot and plume is significant in chemical plume tracing (CPT). In the time immediately following the loss of chemical detection during the process of CPT, Track-Out activities bias the robot heading relative to the upwind direction, expecting to rapidly re-contact the plume. To determine the bias angle used in the Track-Out activity, we propose an online instance-based reinforcement learning method, namely virtual trail following (VTF). In VTF, action-value is generalized from recently stored instances of successful Track-Out activities. We also propose a collaborative VTF (cVTF) method, in which multiple robots store their own instances, and learn from the stored instances, in the same database. The proposed VTF and cVTF methods are compared with biased upwind surge (BUS) method, in which all Track-Out activities utilize an offline optimized universal bias angle, in an indoor environment with three different airflow fields. With respect to our experimental conditions, VTF and cVTF show stronger adaptability to different airflow environments than BUS, and furthermore, cVTF yields higher success rates and time-efficiencies than VTF.

## 1. Introduction

Many animals exhibit the capability of tracing the plume of chemical stimuli to its source using the olfactory sense: Pacific salmons retain odor memories of their home stream to guide homeward migration [1]; crustacean species sense the relatively rare patches of coral reef to search for their settlement habitat [2]; crabs [3] and crayfishes [4] use chemical cues to find the source of food odor; male moths [5] navigate along pheromone plume, which consists of intermittent, wind-blown patches [6] of chemical substances separated by large voids, to locate females, *etc.* Mobile robots capable of such feats (*i.e.*, tracing the chemical plume to its source using the olfactory sense) can be used in sweeping mines, searching for survivors in collapsed buildings, and finding the leakage sites of hazardous chemicals. Compared with living animals trained for similar purposes, robots have the capability of searching in dangerous environments without impairment. In addition, while static sensor nodes [7] deployed for environment monitoring can only cover a limited region, mobile robots can theoretically cover an indefinitely large area. Therefore, mobile robots are more robust to hazards than trained animals and are more flexible than static sensor nodes.

From the early 1990s, various biomimetic methods for chemical plume tracing (CPT) using mobile robots have been proposed. A class of most extensively studied biomimetic CPT methods are the ones imitating the pheromone plume tracing behavior of male moths to search for females [8]. Li *et al.* developed, optimized, and evaluated [9] a moth-inspired cross-plume counterturning strategy, and proposed [10] a behavior-based adaptive mission planner (AMP). Four behaviors were implemented in this AMP: Plume finding, plume tracing, plume reacquiring, and chemical source declaration, in which the second and third behaviors are moth-inspired. Marques *et al.* [11] concluded that the moth-inspired method is more effective than the bacterium *E. coli*’s chemotaxis method. Lilienthal *et al.* [12] proposed a moth-inspired fixed motion pattern which is (re-)started when an increased chemical concentration is sensed. Ishida [13] proposed a moth-inspired “casting” behavior, *i.e.*, cross-wind movement with gradually broadened scanning width, which can be combined with the upwind movement to realize an efficient CPT method. Generally, two distinctive features [14] of moth’s plume-tracing behavior have been replicated in these methods. Firstly, the flow direction while detecting the plume was exploited as a reliable directional cue to approach the females; secondly, counter-turning movements were used as fail-safe mechanisms to reacquire the plume in unsteady environments.

In particular, the AMP proposed in [10] has successfully accomplished the CPT mission over one hundred meters in near-shore ocean environments. Specifically, in the AMP proposed in [10], plume finding behavior is activated at the initial stage of CPT to find the first chemical clue. After the first chemical detection event, plume tracing behavior, which is decomposed into Track-In and Track-Out activities, is activated. Track-In activity steers the robot upwind when it detects the chemical. Track-Out activity, which moves the robot along a biased upwind direction (*i.e.*, the summation of real-time upwind direction and a universal bias angle), is activated immediately after the robot losing contact with the plume, expecting to rapidly re-contact the lost plume. The way of adding a universal bias angle to the real-time upwind direction to form the robot heading in Track-Out activity is referred to as biased upwind surge (BUS) method in the rest of this paper. If Track-Out activity fails to re-contact the plume in a predefined time span, plume reacquiring behavior, which outputs a clover-leaf-shaped route for the robot, is activated as a fail-safe mechanism for further re-contacting the plume and then activating the plume tracing behavior. To declare the chemical source location, chemical source declaration behavior is activated if six successive last detection positions (LDP), *i.e.*, the position where the robot lost contact with the plume, lie close to each other.

The Track-Out activity comprises two successive processes: (1) rotating: At the beginning of the Track-Out activity, the robot rotates to align its heading with the biased upwind direction; (2) sprinting: The robot moves along the biased upwind direction. On one hand, a big acute bias angle along the right direction is often needed for directing the robot towards the lost plume. On the other hand, most real mobile robots (e.g., fin-controlled underwater robots [10], various terrestrial [14,15] or flying [16,17,18] robots) require longer time to rotate a bigger bias angle. To minimize total time spent in the Track-Out activity, a bargain between the time spent in rotating and sprinting can be stricken by properly determining the bias angle. However, the bias angle used in BUS [10] is optimized beforehand for all possible Track-Out activities using offline Monte-Carlo simulations. The offline optimized bias angle would not be optimal when it is used in real environments with airflow field different from the simulated ones. The problem of determining the bias angle to adapt different real airflow fields needs further investigations.

In this paper, we propose an online reinforcement learning (RL) method to determine the bias angle used in Track-Out activities. In the proposed RL method, action-values [19] are generalized from recently stored instances of successful Track-Out activities. Since the structure of stored instance resembles chemical trail (*i.e.*, trail of chemical substances laid on the ground), the proposed RL method, which guides the robot by its previous “trails”, is analogous to chemical trail following [14,20]. Nevertheless, the robot does not lay or follow real chemical trails in our method. Thus, the proposed RL method and the stored instance are referred to as virtual trail following (VTF) method and virtual trail (VT), respectively. The VTF method defers the determination of bias angle till the beginning of each Track-Out activity and learns to steer the robot in an online manner. Thus, it has the merit of adaptation to different real environments which remedies the drawback of offline optimization in BUS. Another merit of the VTF method is that it enables a straightforward solution to realizing collaboration among multiple robots: The robots can share their stored VTs with each other for learning collaboratively [21]. Therefore, we further propose a collaborative VTF (cVTF) method, in which multiple robots store their own VTs, and learn from the stored VTs, in the same database. Finally, we compare VTF, cVTF, and BUS, as well as a reverse BUS (rBUS) method which is used for clarifying the influence of bias angle on BUS, in real-world experiments. The experiments were conducted within two different controlled airflow fields, *i.e.*, mildly and severely fluctuating airflow fields, and a naturally ventilated indoor airflow field.

The rest of this paper is organized as follows: BUS and the AMP proposed in [10], as well as the fundamental of RL, are introduced in Section 2. The VTF and cVTF methods are detailed in Section 3. Experimental setup and results are presented in Section 4 and Section 5, respectively. Conclusions are given in Section 6.

## 2. Background

### 2.1. Track-Out Activity Using BUS

**Figure 1:**
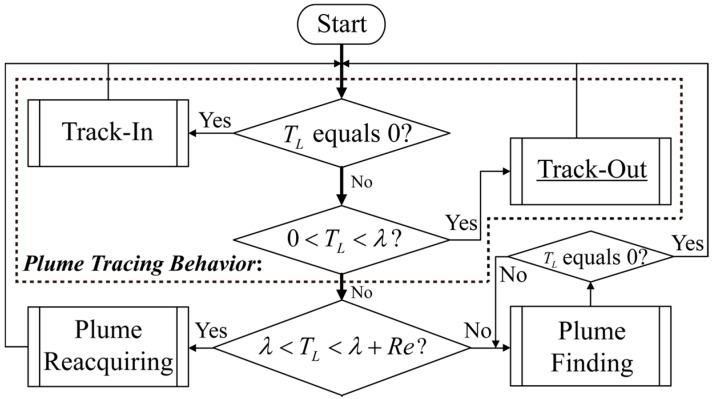
Circulation process following the first chemical detection event.

Before introducing the BUS method, the overall logic of the AMP proposed in [10] is sketched in Figure 1. At the beginning of CPT, the robot is maneuvered by plume finding behavior to find a plume. Once the first chemical detection event occurs, the robot is controlled by the circulation process shown in Figure 1, where $T_{L}$ denotes the number of cycles from the last chemical detection event till the current time; λ and Re are the cycle limit of the Track-Out activity and plume reacquiring behavior, respectively. (Note: A list of the notation used in this paper is given in the Appendix.) As shown in Figure 1, the plume tracing behavior is decomposed into Track-In and Track-Out activities. The activation or inhibition of plume reacquiring behavior, plume finding behavior, as well as Track-In and Track-Out activities, is triggered by determining whether the value of $T_{L}$ falls within the corresponding range or not. Only one activity or behavior is activated at the same time. This circulation process can be terminated by the source declaration behavior, which is not included in Figure 1 because we focus on the behavior of re-contacting the lost plume in this paper.

When $T_{L}$ falls within the range (0,λ], the Track-Out activity is activated. In other words, the Track-Out activity starts when $T_{L}$ equals one, and ends when $T_{L}<\lambda$ if succeeded in re-contacting the plume or when $T_{L}$ equals λ if failed. In BUS, the robot heading θ(k) is calculated as follows (see Figure 6 in [10] and Equation (4) in [9]):

(1) $$\begin{array}{l} \theta (k)=\psi (k)+180{}^{\circ}+\beta (k),\\ \beta (k)=10{}^{\circ}\times \mathrm{sgn}\left(\psi (k)-∠\vec{x(k)x_{L}}\right) \end{array}$$

where ψ(k), β(k), and x(k) are the angle of wind direction, the bias angle, and the robot position at the *k*-th cycle, respectively; the magnitude of β(k), *i.e.*, 10°, is the optimized result obtained using offline Monte-Carlo simulations in [9]; $x_{L}$ and $∠\vec{x(k)x_{L}}$ denote the LDP and the angle of the vector pointing from x(k) to LDP, respectively.

**Figure 2:**
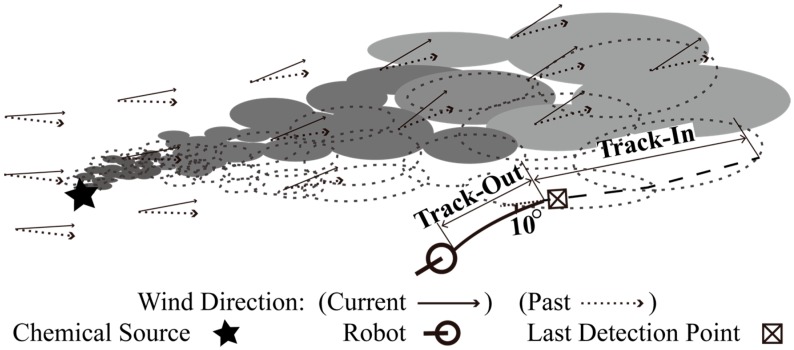
Robot trajectories obtained using BUS in the Track-Out activity. Due to the variation of wind direction, the chemical patches in the plume were carried from their past positions (*i.e.*, dotted ellipses) to current positions (*i.e.*, grey oblong plates).

An illustration of using BUS in a Track-Out activity triggered by the event that time-varying wind blew the plume away from the robot is shown in Figure 2. Unfortunately, BUS steers the robot away from the departing plume in this case, which can be inferred as follows: Suppose that the Track-Out activity begins at the *k*-th cycle and that the anticlockwise direction is positive. Then, ψ(k)>ψ(k−1), since the wind has shifted anticlockwise in Figure 2. Since the robot moves upwind at the (*k* − 1)-th cycle, $∠\vec{x(k)x(k-1)}$ equals ψ(k−1). Moreover, since x(k−1) equals $x_{L}$, $∠\vec{x(k)x_{L}}$ equals ψ(k−1). Thus, $\mathrm{sgn}(\psi (k)-∠\vec{x(k)x_{L}})$ equals sgn(ψ(k)−ψ(k−1)) which is bigger than zero, β(k) and θ(k) equal 10° and ψ(k)+190°, respectively. In addition, ψ(k)+190° is bigger than ψ(k−1)+180° which equals θ(k−1), θ(k)>θ(k−1), which makes the robot turn anticlockwise and move away from the chemical plume.

### 2.2. Reinforcement Learning

The term RL was primitively used for characterizing the problem of learning from interaction between an agent and its environment to maximize the total amount of reward received by the agent over the long run [19]. Any method that is suitable for solving the RL problem can be considered as a RL method. At each learning step, the agent conducts an action a,a∈A in state s,s∈S, then moves to state $s^{\prime },s^{\prime }\in S$ and receives a reward r at the next learning step. The action-value $Q^{\pi }(s,a)$ (*i.e.*, Q-value) defines the expected discounted reward when action a is selected in state s. $Q^{\pi }(s,a)$ is expressed as:

(2) $$Q^{\pi }(s,a)=E_{\pi (s,a)}\left\{\sum_{k=0}^{\infty }\gamma^{k}r_{k+1}|\begin{array}{c} s_{k}=s,\\ a_{k}=a \end{array}\right\}$$

where $r_{k+1}$ represents the reward received at the (*k* + 1)-th learning step; γ∈[0,1] is the discount rate. The RL problem can be solved by finding an optimal policy $\pi^{*}$ which guarantees $Q^{\pi^{*}}(s,a)\geq Q^{\pi }(s,a)$ for all s∈S.

Most action-value-based RL methods follow the idea of generalized policy iteration (GPI) [19] to determine the optimal policy. A GPI consists of two interacting processes: Policy evaluation and policy improvement. The former calculates the Q-value function using the current policy, while the latter makes the policy greedy with respect to the original value function. In the popular Q-learning algorithm [22], policy evaluation is realized according to:

(3) $$\begin{array}{l} Q_{k+1}(s,a)=Q_{k}(s,a)\\ \text{ +}\alpha [r_{k+1}+\gamma \underset{a^{\prime }\in A}{\max}Q_{k}(s^{\prime },a^{\prime })-Q_{k}(s,a)] \end{array}$$

where α∈[0,1] denotes learning rate. Then, policy improvement is performed using the ε-greedy policy [19], which selects the action with the highest Q-value with the probability 1−ε or randomly selects an action otherwise.

In tabular RL problems [19], the Q-learning algorithm has been proved to be convergent when each state-action pair is visited indefinitely often. When the state and action spaces are very huge or continuous, tabular methods would suffer from the curse of dimensionality. It is impossible to maintain an individual update of Q-value for every state-action pair in continuous state spaces [23]. Q-value approximation, which approximates the Q-values in states that have not been experienced before using previously obtained learning results [23], appears to be a feasible technique to handle continuous state and action spaces. In principle, any of the methods studied in function approximation, e.g., artificial neural network, locally weighted regression [24], and decision-trees, can be used in RL [19]. At present, the convergence proof of the RL methods with Q-value approximation is lacking. Nevertheless, no matter how complex about the convergence, there still have been a lot of works about combining Q-value approximation with RL methods in continuous state and action spaces [23,25], since they promisingly provide good solutions even not optimal ones.

## 3. Learning to Re-Contact the Plume via VTF and cVTF

### 3.1. VTF Method

First, some preliminaries, including the problem formulation, and handling of the continuous action and state spaces, are presented. Then, two main steps of VTF, *i.e.*, policy improvement and policy evaluation, are detailed.

#### 3.1.1. Preliminaries

##### Problem Formulation

As mentioned, the Track-Out activity is realized by rotating the robot to align its heading with a new heading angle, and then move ahead. The new heading angle in VTF is represented as follows:

(4) $$\begin{array}{l} \theta =\psi_{L}+\beta ,\\ \beta \in [-\bar{\beta },\bar{\beta }],\bar{\beta }>90{}^{\circ} \end{array}$$

where $\psi_{L}$ denotes the wind direction measured at $x_{L}$; $-\bar{\beta }$ and $\bar{\beta }$ are the lower and upper bounds of the bias angle β, respectively. The constraint that $\bar{\beta }>90{}^{\circ}$ is used to avoid the robot moving towards the downwind area of $x_{L}$, which could steer the robot away from the chemical source and deteriorate the overall time-efficiency of CPT.

The problem of determining β in Equation (4) to minimize the time spent in an individual Track-Out activity is formulated as a RL problem. In this RL problem, each Track-Out activity corresponds to an individual learning step, which usually extends over multiple cycles. At the beginning of each learning step, the robot starts from one position, rotates, moves, and then arrives at another position at the end of the learning step. State is defined as the robot position, so the start state s and end state $s^{\prime }$ correspond to the start and end positions, respectively. Action $a_{i}$ is defined as rotating to and then moving along the direction with angle $\theta_{i}=\psi_{L}+\beta_{i}$. Possible robot positions and values of β are mapped one-to-one with the states and actions, respectively. Thus, the continuous spans of robot position and β lead to continuous state and action spaces, respectively.

At the end of each learning step, the robot receives a numerical reward, which is defined as $r=\lambda -T_{L}$. The reward is inversely proportional to the time spent in the learning step, *i.e.*, $T_{L}$. Thus, maximizing the Q-value, *i.e.*, expected total rewards, reflects the objective of learning, *i.e.*, rapidly re-contacting the lost plume.

##### Handling of the Continuous State and Action Spaces

To handle the above-mentioned continuous state space, Q-value is generalized from stored VTs using a locally weighted average (LWA) method. The VT is represented as a structure $<s,s^{\prime },Q(s,a)>$, where Q(s,a) is the Q-value of conducting a in state s which results in the associated VT. Thus, the dot operator is used to represent the elements of a VT (e.g., u represents a VT, then u.s is the start state of u) in the rest of this paper.

**Figure 3:**
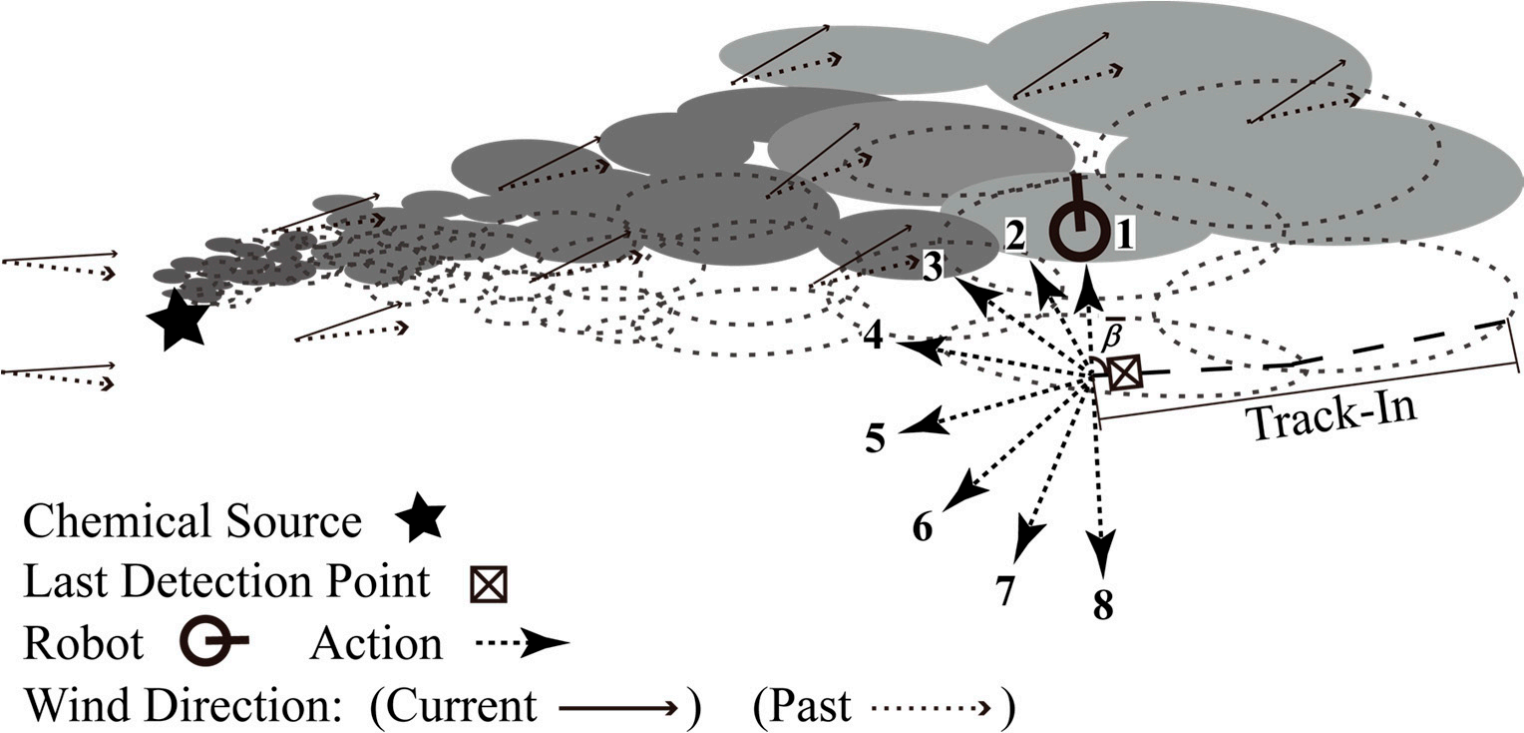
Discretizing the continuous action space to a set of eight actions.

The continuous action space is handled by discretizing the continuous span $\left[-\bar{\beta },\bar{\beta }\right]$ to the set:

(5) $$\left\{\beta_{i}=\bar{\beta }-\frac{2\bar{\beta }}{M-1}\cdot (i-1),\;i=1,2,..,M\right\}$$

where M denotes the number of actions. To avoid $\beta_{(M+1)/2}$ and θ equal zero and $\psi_{L}$ respectively, which in turn make the robot continue moving upwind in the Track-Out activity, M is set as an even integer. Figure 3 illustrates the case that $\bar{\beta }$ and M equal 90° and eight, respectively.

#### 3.1.2. Main Steps of the VTF Method

**Figure 4:**
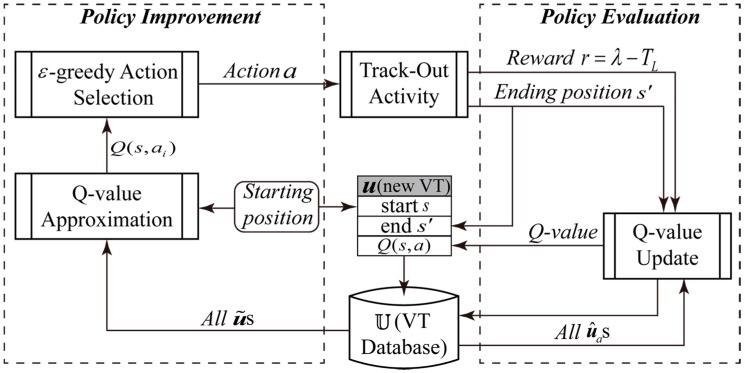
Flow chart of the VTF method. $\tilde{u}$s and ${\hat{u}}_{a}$s denote the nearby VTs of u and the VTs associated with action a, respectively.

**Figure 5:**
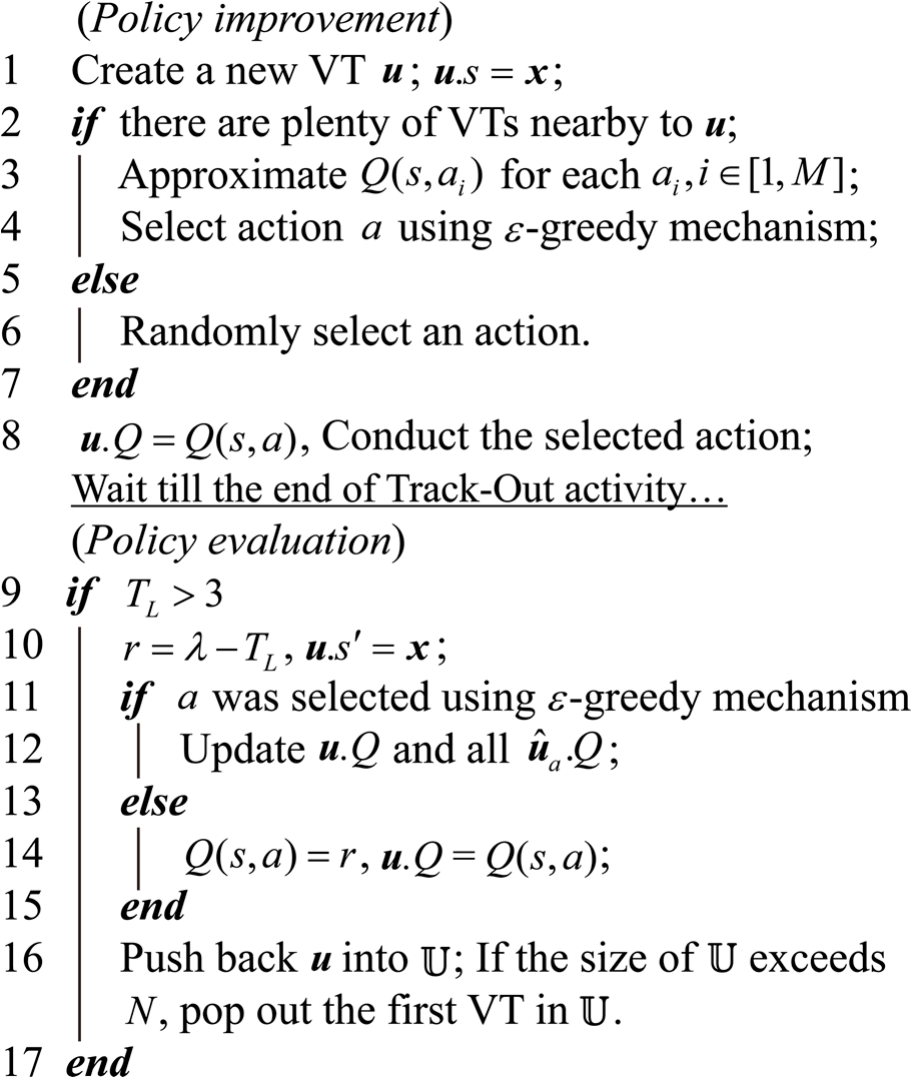
Pseudo-code of the VTF method. ${\hat{u}}_{a}$ denotes the VT associated with the action a.

The flow chart and pseudo-codes of the VTF method are shown in Figure 4 and Figure 5, respectively. Each learning step comprises two main steps: Policy improvement and policy evaluation, which are conducted at the beginning and the end of Track-Out activities, respectively.

##### Policy Improvement

The process of policy improvement includes the steps enclosed in the left dashed frame of Figure 4, which corresponds to lines 1–7 in Figure 5. Policy improvement takes the robot position and stored VTs as input, and outputs a selected action a for the corresponding Track-Out activity.

**Figure 6:**
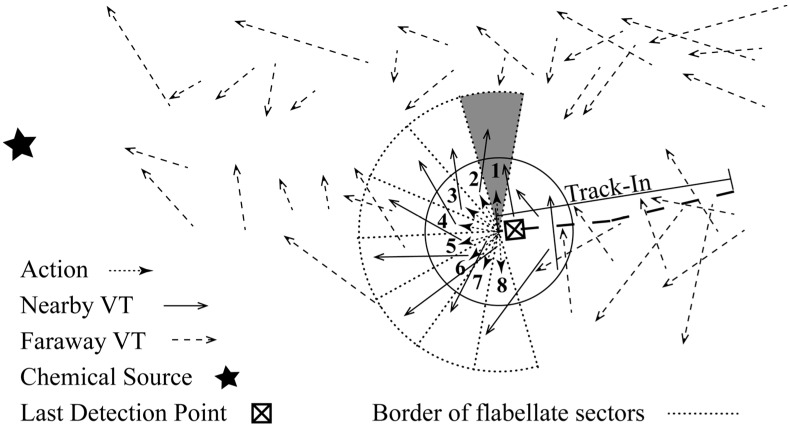
Q-value approximation based on nearby VTs. The solid circle encloses the neighboring area of the starting state in the following Track-Out activity, *i.e.*, u.s.

At first, a new VT, denoted as u, is created, and the robot position is set as u.s. Then, the ε-greedy mechanism is used to determine the output action a (line 4 in Figure 5). To determine $\max_{a}Q(s,a)$ and $a_{*}$ in the ε-greedy mechanism, $Q(s,a_{i}),i\in [1,M]$ are approximated using the LWA method [24] (line 3 in Figure 5). The LWA method has the property of emphasizing relevant data points. In our problem, VTs are the data points, and the distance between two VTs measures their relevance. The distance between two VTs is defined as the distance between their start states. If the start state of a VT falls within the neighbourhood of u.s, *i.e.*, a disk-shaped area centred at u.s with radius of $d_{th}$, the VT is considered as a nearby VT of u. As shown in Figure 6, the neighbourhood of u.s is represented as a disk with solid edge. 

The LWA-based Q-value approximation method consists of three steps:

(1) Find nearby VTs of u in the database, which are denoted as $\tilde{u}$s. As mentioned, Q-value is approximated based on VTs that are previously stored in a VT database U. In Figure 6, nearby and faraway VTs are represented as solid and dashed arrows, respectively.
(2) Associate the nearby stored VTs with the M actions. Suppose that $a_{i}$ covers a flabellate sector bi-partitioned by $a_{i}$. In Figure 6, the flabellate sector covered by $a_{1}$ is marked as shadowed. The radius and included angle of the flabellate sector are $\lambda \cdot v_{\max}$ ($v_{\max}$ is the maximal velocity of the robot) and $2\bar{\beta }/\left(M-1\right)$, respectively. Then, if the end state of $\tilde{u}.s^{\prime }$ falls within the sector covered by $a_{i}$, $\tilde{u}$ is associated with $a_{i}$. The VT associated with $a_{i}$ is denoted as ${\hat{u}}_{i}$. In Figure 6, there are two VTs associated with $a_{1}$, while there is only one VT associated with each of other actions.
(3) Approximate $Q(s,a_{i})$ by weighted-averaging the Q-value of all ${\hat{u}}_{i}$s. The weight for the Q-value of the *j*-th ${\hat{u}}_{i}$ (*i.e.*, ${\hat{u}}_{ij}.Q$), which is denoted as $w_{ij}$, is calculated as:
  (6) $$\begin{array}{l} Q(s,a_{i})=\sum_{j}\left(Q_{ij}\cdot w_{ij}\right)\\ w_{ij}=\frac{K(d(s_{ij},s))}{\sum_{j}K(d(s_{ij},s))}\\ K(d(s_{ij},s))=\frac{1}{1+d(s_{ij},s)} \end{array}$$
  where $s_{ij}$ and $Q_{ij}$ are the start state and the Q-value of ${\hat{u}}_{ij}$, respectively; $d(s_{ij},s)$ is the distance between $s_{ij}$ and s.

Note that, at the early stage of CPT, there are only a small number of VTs in U. If the number of nearby VTs is less than M, which means there are not enough nearby VTs to be used in the LWA-based Q-value approximation method, then an action is randomly selected from the set of M actions (see line 6 in Figure 5).

##### Policy Evaluation

The process of policy evaluation includes the steps enclosed in the right dashed frame of Figure 4, which corresponds to lines 8–14 in Figure 5. When the Track-Out activity ends, policy evaluation process takes the end state and the time spent in the Track-Out activity (*i.e.*, $s^{\prime }$ and $T_{L}$) as inputs, and outputs the updated Q-values of the conducted action a and the VTs associated with a.

u.Q is evaluated using Equation (3), in which $\underset{a^{\prime }}{\max}Q(s^{\prime },a^{\prime })$ is determined in the same way as determining $\underset{a}{\max}Q(s,a)$. In addition, the Q-value of the VT associated with the conducted action a is evaluated as follows:

(7) $$\begin{array}{l} {\hat{u}}_{a}.Q={\hat{u}}_{a}.Q+\\ \;w\cdot \alpha [r+\gamma \underset{a^{\prime }\in A}{\max}Q(s^{\prime },a^{\prime })-{\hat{u}}_{a}.Q] \end{array}$$

where ${\hat{u}}_{a}$ is one of the VTs associated with action a, w is the associated weight. Compared with Equation (3), an additional weighting factor w is additionally utilized in Equation (7) to control the extent to which ${\hat{u}}_{a}.Q$ should be varied. Recall that the VTs associated with action a have been determined in LWA-based Q-value approximation conducted at the beginning of the Track-Out activity. Moreover, a weight that is positively related to the distance between ${\hat{u}}_{a}$ and u has been calculated using Equation (6). These weights are reused in Equation (7) so that the variation of ${\hat{u}}_{a}.Q$ is positively correlated with the distance between ${\hat{u}}_{a}$ and u.

Finally, u is pushed into the U. If the size of U exceeds N, the oldest VT in U is popped out. This kind of first-in-first-out configuration can adapt the stored VTs to dynamic environments.

### 3.2. Collaborative VTF Method

On the premise that VTF is utilized as the strategy of Track-Out activity by multiple robots for CPT in the same field, cVTF is realized by sharing a common VT database among these robots:

(1) During policy improvement, the VTs in the same database are exploited by multiple robots in the LWA-based Q-value approximation. In other words, the robots determine their own heading by learning from the experience of each other at the beginning of Track-Out activities.
(2) The Q-value of nearby VTs stored in the same database are updated by multiple robots. Moreover, the VTs generated by multiple robots are pushed into the same database after policy evaluation.

## 4. Experimental Setup

In this section, the real mobile robots, experimental scenarios, and experimental schemes are introduced. Moreover, the process of selecting parameters for the methods is detailed. The proposed VTF and cVTF methods were compared with BUS and rBUS (see Section 4.3 for details) in real-world experiments using multiple robots. Since cVTF involves collaboration among multiple robots, four real mobile robots were used to conduct a multi-robot CPT mission in our experiments. Although VTF is capable of working with a single robot, running it on multiple robots independently can include the influence of obstacle avoidance and enable an equitable comparison between VTF and cVTF. So do BUS and rBUS.

### 4.1. Real olfactory robots

**Figure 7:**
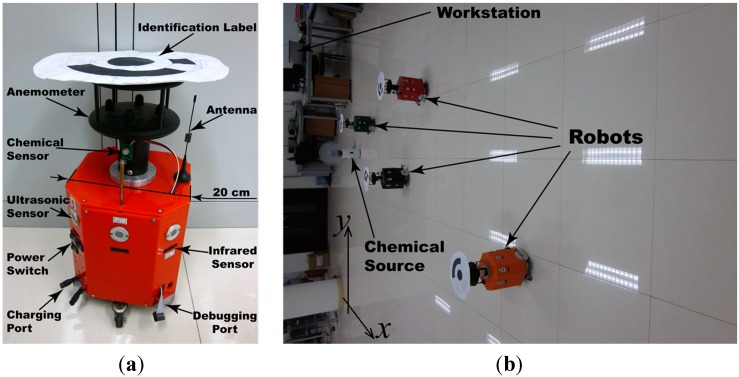
Mobile olfactory robots used in the experiments. (**a**) One of the MrCollie robots; (**b**) a scene of controlling the robots in the experiments.

The mobile olfactory robots used in our experiments, namely MrCollie [26,27], are displayed in Figure 7. A chemical sensor (MICS-5521, SGX Sensor Technology, Co. Ltd.: Neuchatel, Switzerland) is sustained on top of the case by a pillar. Eight ultrasonic sensors and eight infrared sensors are mounted around the case to detect the remote (0.8~3 m) and close (0~0.8 m) obstacles, respectively. On the top of the robot, an anemometer (WindSonic, Gill Instruments, Co. Ltd.: Hampshire, UK) is mounted for measuring real-time wind velocity. A hard-wired CCD camera is mounted on the ceiling over the valid search region to capture the image of identification labels stuck on the top of the anemometers. By processing the acquired image on a workstation, the orientation, index, and global position of the robots can be recognized. The workstation received real-time measurements from the robots, conducted the CPT methods for about two cycles per second, and sent movement commands back through ultra-high-frequency radio waves.

Referring to [17] and [28], chemical detection event was determined by comparing the transient concentration measurement [29] c(k) with an adaptive threshold $\bar{c}(k)$: $c(k)>\bar{c}(k-1)$ and $c(k)\leq \bar{c}(k-1)$ indicate the detection and non-detection event at the *k*-th cycle, respectively. The adaptive threshold $\bar{c}(k)$ proposed in [28] was defined as:

(8) $$\bar{c}(k)=\{\begin{array}{ll} \delta \cdot \bar{c}(k-1)+(1-\delta )c(k), & \;\;k\geq 0\\ \;\;c(k), & k=0 \end{array}$$

where δ was set to 0.5 [17,28]. Besides, due to the intermittent feature of real chemical plumes, short-term flashed non-detection events would occur when the robots get into the voids between chemical patches within the plume. Thus, to preclude this case, contact between the robot and plume was considered as lost after two consecutive non-detection events. In other words, the Track-Out activity was activated when $T_{L}$ equals three in our experiments.

To obtain absolute wind velocities, the robots’ theoretical velocities were subtracted from the relative wind velocities, which were measured with a sampling period of 0.5 s by the anemometer. Moreover, to reduce measurement errors, the absolute wind velocities were moving-averaged across two seconds before being used in our experiments. The feasibility of calculating the absolute wind velocities based on the robots’ theoretical velocities is analyzed in the appendix.

### 4.2. Experimental Scenarios

**Figure 8:**
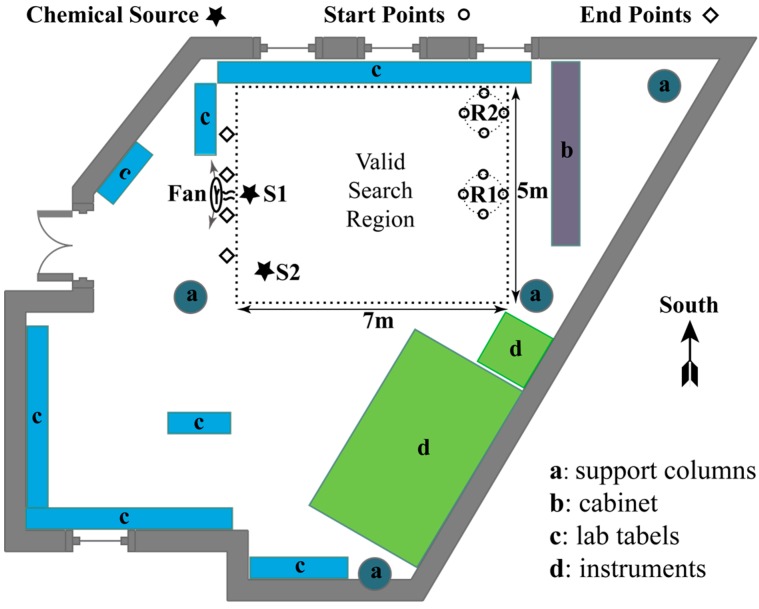
Plan sketch of the laboratory. The valid search region is represented as a rectangular area with dotted edges.

Experiments were carried out in a laboratory, in which the valid search region is a 5 m × 7 m rectangular area, as shown in Figure 8. An ultrasonic humidifier, which can spray atomized ethanol vapour out from its nozzle, was used as the chemical source. Experiments were conducted in three different airflow fields, including two controlled airflow fields and one naturally ventilated airflow field. These airflow fields were constructed as follows:

- Two controlled airflow fields: With the door and all windows of the laboratory closed, mildly and severely fluctuating wind were produced by oscillating the fan with scopes of about 30° and 90°, respectively. In these two controlled airflow fields, the chemical source was placed at S1, and the robots started from R1.
- Naturally ventilated airflow field was constructed by opening the windows and the door of the laboratory in a windy day. The chemical source was placed at S2, so that the released chemical can be blown by the wind coming from the door and the window in the bottom wall. The robots started from R2.

### 4.3. Experimental Scheme

Because this paper focused on re-contacting the lost plume, the plume finding and source declaration processes were omitted in our experiments. The resulting control logic used in our experiments is as follows: At the start of a CPT run, each robot waited at its start point till it detected an above-threshold concentration measurement. After the first chemical detection event, the robot was controlled by the circulation process shown in Figure 1, in which the time constraint for plume reacquiring behaviour was removed, *i.e.*, Re=+∞. The activation or inhibition of the robots’ behaviors or activities was triggered independently based on the value of their own $T_{L}$. Once a robot got into the neighbourhood of the chemical source, where the distance between the robot and chemical source did not exceed $d_{th}$, it was steered to its end point. When all robots arrived at their end points, the CPT run ended.

The upwind movement [10] and the “casting” behaviour [13,14] were used as the Track-In activity and the plume reacquiring behaviour, respectively. Four alternative methods were employed in Track-Out activities: BUS, rBUS, VTF, and cVTF. BUS, VTF, and cVTF have been detailed in Section 2.1, Section 3.1 and Section 3.2, respectively. rBUS was realized by determining the robot heading during Track-Out activity as follows:

(9) $$\begin{array}{l} \theta (k)=\psi (k)+180{}^{\circ}+\beta (k),\\ \beta (k)=10{}^{\circ}\times \mathrm{sgn}\left(∠\vec{x(k)x_{L}}-\psi (k)\right) \end{array}$$

where the bias angle β(k) is the opposite number of the bias angle used in Equation (1). In the typical case shown in Figure 2, where the wind shifted anticlockwise and blew the plume away from the robot, BUS made the robot turn anticlockwise and move away from the plume. It was not clear that whether the performance of BUS is dominated by the sign of bias angle used in Equation (1) or not. Thus, BUS was compared with rBUS, which can make the robot turn clockwise in expectation of chasing the departing plume in the case shown in Figure 2.

The artificial potential field (APF) based method proposed in [30], which took relative position and velocity of moving obstacles into account, was used in our experiments for avoiding moving obstacles (*i.e.*, other robots). In the APF-based obstacle avoidance method, the robot is attracted to its goal position $x_{g}=\{x_{g},y_{g}\}$, whereas repulsed away from nearby obstacles. Two types of movements were realized in our experiments:

(1) Moving along a designed direction (e.g., upwind direction in Track-In activities, the direction learned in Track-Out activities): $x_{g}$ was set to a position in front of the robot along the designed direction. To move the robot at x={x,y} along direction θ, for example, the goal position $x_{g}$ was set to:
  (10) $$\{\begin{array}{c} x_{g}=x+d_{big}\cdot \cos\theta \\ y_{g}=y+d_{big}\cdot \sin\theta \end{array}$$
  where $d_{big}$ should be big enough to make sure the APF method outputs sufficient attractive force for the robot.
(2) Cross-wind movement with gradually broadened scanning widths in the “casting” behaviour [13]: Suppose the robot position at the beginning of “casting” was x={x,y}. During the “casting” behavior, the robot was moved towards $x_{g}$. Once the robot arrived at the old position of $x_{g}$, $x_{g}$ was reset as follows:
  (11) $$\{\begin{array}{c} x_{g}=x\\ y_{g}=y+{\left(-1\right)}^{n_{t}}\cdot \mathrm{sgn}(y_{L}-y)n_{t}d_{ss} \end{array}$$
  where $y_{L}$, $n_{t}$, and $d_{ss}$ are the y-coordinate of $x_{L}$, the number of times that the robot has arrived at $x_{g}$, and the scanning span added to the scanning width, respectively. Note that the resulting robot trajectories do not strictly equal the one illustrated in [13] and [14]. Nevertheless, plume reacquiring behaviour is not the main concern of this paper.

### 4.4. Parameter Selection

Three categories of parameters were used in our methods:

(1) Common parameters of Track-Out activity: λ and δ, which influence the performance of all methods used in the Track-Out activity. The value of δ was set to 0.5 in [17,28], which both used the adaptive concentration threshold in Equation (8) to determine chemical detection events.
(2) Parameters for RL: ε, γ, and α. In an analogous continuous instance-based Q learning method [25], ε, γ, and α were set to 0.01, 0.9, and 0.1, respectively.
(3) Parameters for obstacle avoidance using the APF method: $v_{\max}$, $d_{th}$, $d_{big}$, and $d_{ss}$, which were set to 15 cm/s, 45 cm, 4 m, and 80 cm, respectively. The guideline for selecting these parameters is that the robots would not collide with each other while searching in the valid search region.

The process of selecting the parameters in the first and second categories are detailed in Section 4.4.1 and Section 4.4.2, respectively. The value of the parameters in the third category were not varied in our experiments, because we found they worked quite well in our experiments.

#### 4.4.1. Selecting the Common Parameters of Track-Out Activity

Due to the similar principles underlying BUS and rBUS, as well as underlying VTF and cVTF, only BUS and cVTF were tested for selecting δ and λ. During the process of selecting δ and λ, the second category of parameters (*i.e.*, ε, γ, and α) were set to their old values used in [25].

**Figure 9:**
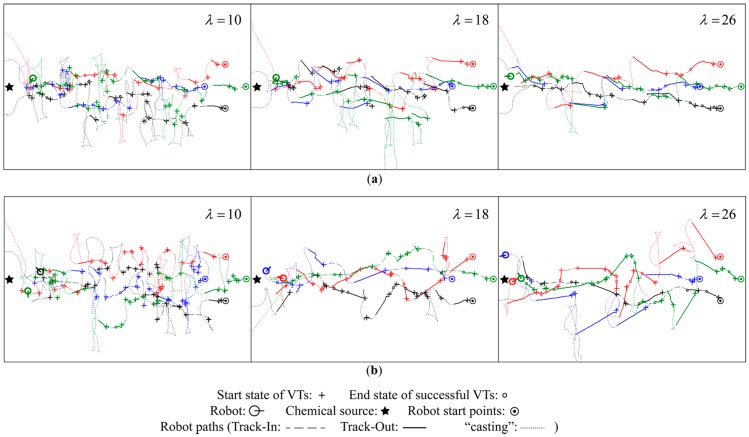
Comparison of the robot trajectories obtained by setting different values of λ. (**a**) Robot trajectories obtained using BUS; (**b**) Robot trajectories obtained using cVTF.

First, δ was kept invariant as 0.5, while the value of λ was set to 10, 18, and 26, which corresponds to a maximal period of 4, 8, and 12 s for the Track-Out activity. The resulting robot trajectories obtained in individual Track-Out activities are shown in Figure 9.

If a small λ (e.g., λ=10) is used, the robot failed to re-contact the plume mostly because it only sprinted for a short span away from the LDP. Therefore, the value of λ was set to 18 in the rest of this paper. As shown in the left sub-figures of Figure 9, the robots spent most the time of Track-Out activity for rotating when λ was set to 10, which corresponds to a time of five seconds. However, a large λ (e.g., λ=26) brings about large failure costs when Track-Out activities fail to direct the robots towards the lost plume. In the right sub-figures of Figure 9, many failed Track-Out activities steered the robots far away from the LDP in vain. Therefore, we select a medium case and set λ to 18 in the rest of this paper.

**Figure 10:**
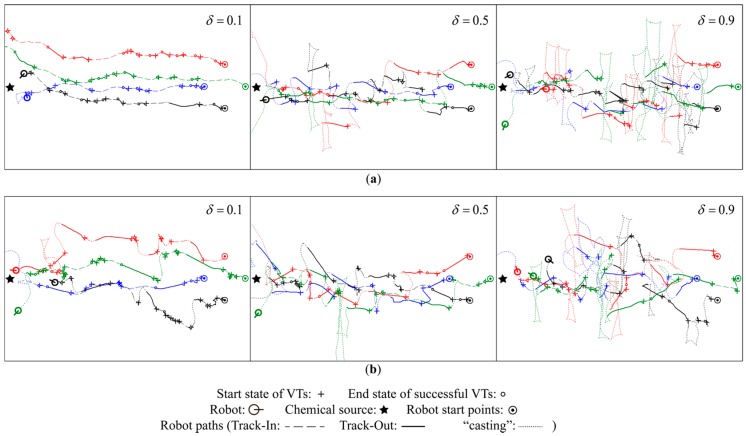
Comparison of the robot trajectories obtained by setting different values of δ (**a**) Robot trajectories obtained using BUS; (**b**) Robot trajectories obtained using cVTF.

Then, the value of δ was set to 0.1, 0.5, and 0.9. The resulting robot trajectories are shown in Figure 10. Due to the slow recovery time of the MiCS-5521 sensors, both the chemical detection and non-detection events could lag significantly [28] if a fixed concentration threshold was used to determine the chemical detection event. Consequently, the smaller the fixed threshold, the greater the chance of false positive detection (*i.e.*, chemical detection events still occurs even though the robot does not contact the plume); the larger the fixed threshold, the bigger the risk of false negative detection (*i.e.*, failed to detect the chemical contact). The adaptive concentration threshold in Equation (8) can be used to correctly capture a sequence of chemical detection and non-detection events [17,28].

However, if δ in the adaptive concentration threshold is too small, false positive detection events still occurs frequently. As shown in the left sub-figures of Figure 10, the success rates of Track-Out activities were abnormally high when δ was set to 0.1. In particular, the red and green robots were misled to get across the chemical source by the false positive detection events in the upper-left sub-figure. Conversely, the probability of false negative detection is very high if δ is too big. In the right sub-figures of Figure 10, most Track-Out activities failed in re-contacting the lost plume, even in some cases the robots have got close to the chemical source, when δ was set to 0.1. A medium case that setting the value of δ to 0.5 accords with the rule of thumb that chemical detection events occurs more frequently near the chemical source than other places. Therefore, the value of δ was set to 0.5 in the rest of this paper.

#### 4.4.2. Selecting the Parameters for RL

**Figure 11:**
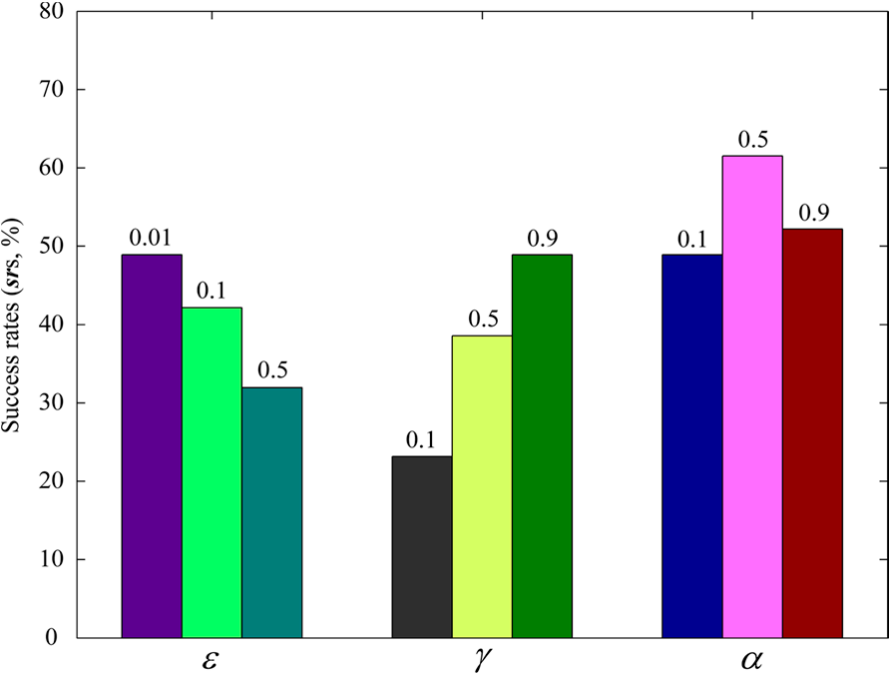
Success rates of the Track-Out activities in ten CPT runs obtained using different values of ε, γ, and α. The tested values are displayed on top of the bars.

Similarly, due to the similar underlying principles of VTF and cVTF, only cVTF was tested. A set of three different values were tested in cVTF for each of ε, γ, and α. Unlike the common parameters of Track-Out activity, which directly influence the activation and inhibition of Track-Out activities, ε, γ, and α only indirectly influence the performance of cVTF through Q-value of the VTs stored in U. The influence of ε, γ, and α on the performance of cVTF are not discernible in the robot trajectory of individual Track-Out activity. Therefore, success rate (*sr*) of the Track-Out activities in ten CPT runs conducted in the severely fluctuating wind field, *i.e.*, the percentage of Track-Out activities in which the robots successfully re-contacted the plume within the cycle limit, was used as the criterion for selecting ε, γ, and α. While comparing different settings of an individual parameter, the value of the other two parameters were kept invariant as those used in the continuous Q-learning method proposed in [25]. For example, while selecting α, the value of γ and ε were set to 0.9, and 0.01, respectively. The *sr*s obtained using different settings of ε, γ, and α; are shown in Figure 11.

As shown in Figure 11, the value of ε, γ, and α that yielded the highest *sr*s among the corresponding set of values are 0.01, 0.9, and 0.5, respectively. Increasing the value of ε and decreasing the value of γ both reduced the *sr*, while a medium value of α yielded the highest *sr.* The extreme low value of ε (*i.e.*, ε=0.01) means that only a very small proportion of exploration is needed for determining the output actions in the policy improvement process of cVTF. A high value of γ stresses future rewards [19]. Figure 11 shows that striving for long-term rewards is important for re-contacting the plume using cVTF. A medium value of α means that both recently acquired rewards and the stored Q-values should be taken into account during the learning process. Based on the results shown in Figure 11, in the rest of this paper, the value of ε, γ, and α were set to 0.01, 0.9, and 0.5, respectively.

## 5. Results and Discussion

In each of the three airflow fields mentioned in Section 4.2, we conducted a group of forty CPT runs, in which each of BUS, rBUS, VTF, and cVTF was tried for ten CPT runs. At the beginning of each CPT run, the databases for storing VTs were cleared. The groups of experiments conducted in mildly fluctuating, severely fluctuating, and naturally ventilated airflow fields were denoted as M group, S group, and N group, respectively. Results obtained in these three groups were presented and discussed in Section 5.1 and Section 5.2, respectively.

### 5.1. Experimental Results

#### 5.1.1. Success Rates

**Figure 12:**
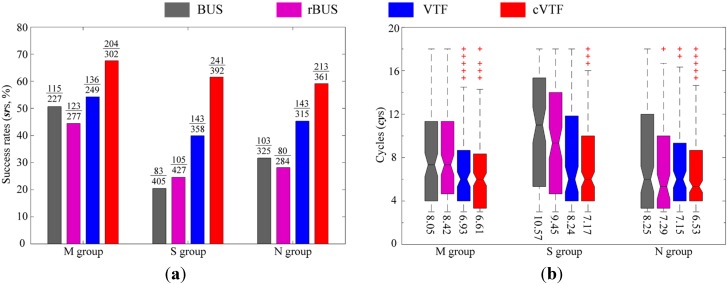
Numerical results. (**a**) Success rates in the three groups of experiments. The numerator and denominator of the fraction on each bar are the corresponding number of successful Track-Out activities and total number of Track-Out activities, respectively; (**b**) Box plots with the whisker lengths specified as 1.0 times the interquartile range for each method in the three groups.

The *sr*s obtained in the three groups are shown in Figure 12a. In addition, the number of successful Track-Out activities and total number of Track-Out activities in each group are displayed as numerator and denominator in the fraction on top of the corresponding bar, respectively.

Firstly, cVTF yielded the highest *sr*s in all groups. The *sr*s of BUS and rBUS are similar in each group, indicating the low *sr*s of BUS are not caused by the sign of bias angle. Secondly, the *sr*s are higher in M group than in S and N groups, averaged across all methods. This indicates that the *sr*s of Tack-Out activities depend on the wind fluctuation. Moreover, the *sr*s obtained by the same method in S and N groups are similar, suggesting that the constructed airflow fields have not been intentionally optimized for the proposed methods. Thirdly, BUS and rBUS cannot adapt to different airflow fields: They yielded noticeably higher *sr*s in M group than in S and N groups, while the difference of *sr*s obtained by VTF or cVTF in different groups are much milder.

#### 5.1.2. Time-Efficiency

Time-efficiency is assessed using the number of cycles that a method was performed per successful Track-Out activity, which is denoted as *cy*s. Recall that each Track-Out activity usually extends over multiple cycles. The larger the *cy*s, the longer the time used to re-contact the plume and the lower the time-efficiency. Box plots on the *cy*s in the three groups are shown in Figure 12b, where the average of *cy*s in each group is displayed on the bottom of the corresponding box plot.

Generally, cVTF yielded the highest time-efficiency, while the time-efficiencies of BUS and rBUS are lower than VTF and cVTF: The average *cy*s of BUS, rBUS, VTF, and cVTF across all groups are 8.96, 8.39, 8.02, and 7.36, respectively. Moreover, the *cy*s of BUS and rBUS are more diverse than those of VTF and cVTF, across different groups or within individual groups: (1) the median of box plots varies more severely in different groups for BUS and rBUS than for VTF and cVTF. For example, the median of box plots in different groups for BUS are 7.33 (M group), 11 (S group), and 6 (N group), while those for cVTF are 6 (M group), 7.33 (S group), and 6.67 (N group). (2) In general, the interquartile range of box plot, which measures the diversity of *cy*s in a single group, averaged across different groups, for BUS and rBUS are larger than those for VTF and cVTF. This indicates that VTF and cVTF are generally more reliable than BUS and rBUS for rapidly re-contacting the plume in CPT.

#### 5.1.3. Robot Trajectories

##### Qualitative Analysis

**Figure 13:**
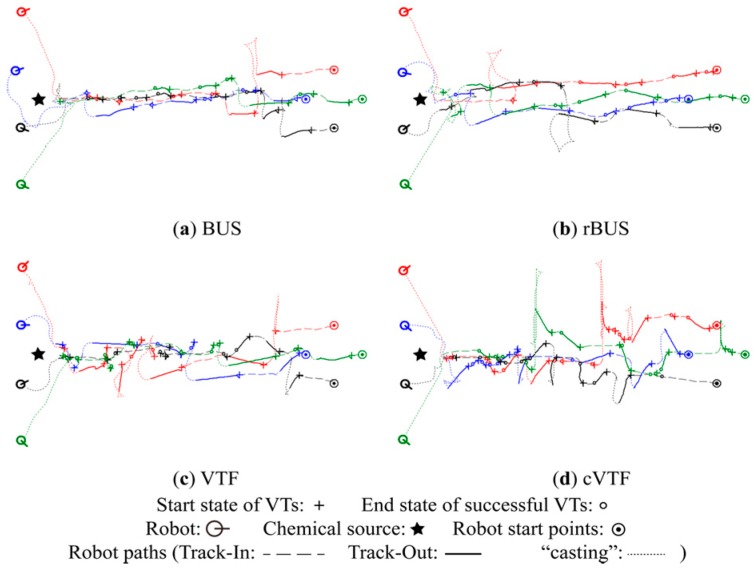
Typical robot trajectories obtained in M group.

**Figure 14:**
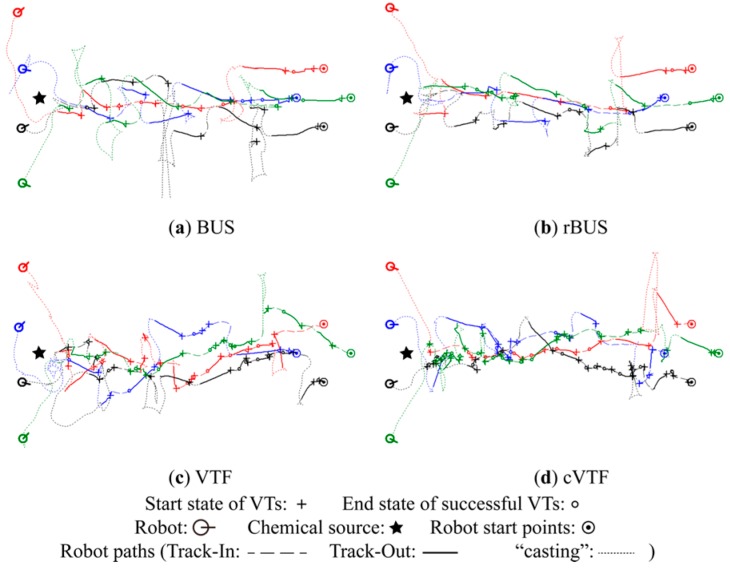
Typical robot trajectories obtained in S group.

**Figure 15:**
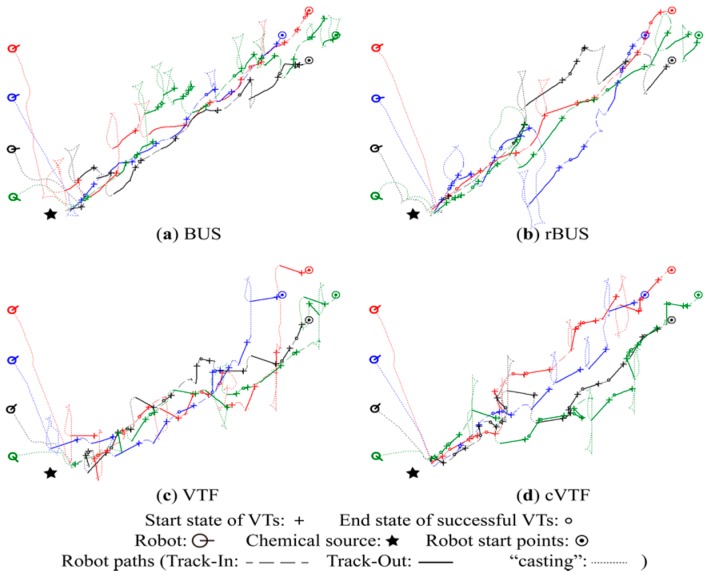
Typical robot trajectories obtained in N group.

Robot trajectories of typical experiments in the M, S, and N groups are shown in Figure 13, Figure 14 and Figure 15, respectively. The winding feature of these robot trajectories is attributed to the APF-based obstacle avoidance algorithm. A video of these typical experiments can be found via the link: http://youtu.be/youhdIpp2kA.

From Figure 13, Figure 14 and Figure 15, it is readily seen that cVTF succeeded more times in each group than other methods, as well as that BUS and rBUS succeeded more times in M group than in S and N groups. Both results accord with the statistical success rates shown in Figure 12a. Most of the robot trajectories resulted from BUS and rBUS are obviously winding, while the trajectories obtained by VTF and cVTF are generally straight (exceptions are caused by obstacle avoidance or the visual-based robot positioning system). Because a fixed universal bias angle and the time-varying wind direction are incorporated into the robot heading in BUS and rBUS, while the robot heading is calculated and then fixed at the beginning of each Track-Out activity in VTF and cVTF. In addition, it is easy to distinguish that VTF and cVTF yielded generally larger bias angles in S and N groups than in M group. This is mainly because the actions with large bias angle were prone to receive higher rewards in S and N groups than in M group.

##### Quantitative Analysis

A new quantitative metric analogous to the distance overhead [31] is used to quantitatively analyze the robot trajectories with respective to individual Track-Out activities. The new metric, namely re-contact distance overhead (*rdo*), is defined as the travelled distance divided by the result of subtracting the distance between the chemical source and the end position of the Track-Out activity from the distance between the chemical source and the start position of the Track-Out activity. For simplicity, travelled distance is approximated as the distance between the start and end positions of the Track-Out activity. The averages of the *rdo*s of successful Track-Out activities in the typical experiments shown in Figure 13, Figure 14 and Figure 15 are given in Table 1.

**Table 1:** Averaged re-contact distance overheads of successful Track-Out activities in the qualitatively analyzed typical experiments.

	BUS	rBUS	VTF	cVTF
M group	1.0162	1.0178	1.2474	1.2647
S group	1.0116	1.0184	1.4782	1.5280
N group	1.0201	1.0193	1.6045	1.6893

In general, VTF and cVTF yielded higher *rdo*s, which mean lower efficiencies with respect to robot trajectories, than BUS and rBUS in all the three groups. While BUS and rBUS yielded similarly low *rdo*s in all groups, the *rdo*s for VTF and cVTF in the S and N groups are higher than those in the M group. This circumstance reflects that the *rdo*s are dependent on the bias angle. The small universal bias angle of ten degree utilized by BUS and rBUS yielded similarly low *rdo*s for the two methods in all groups. As shown in Figure 13, Figure 14 and Figure 15, the bias angles learned by VTF and cVTF are larger in S and N groups than in M group, which accounts for the higher *rdo*s in S and N groups than in M group for VTF and cVTF.

### 5.2. Discussion

In our experiments, BUS and rBUS have achieved much higher *sr*s and time-efficiencies in M group than in S and N groups, which means the optimal bias angle obtained using Monte-Carlo simulation cannot adapt to different real environments. It is assumed that the sign of bias angle dominates the poor performance of BUS, since the robot is steered away from departing plume in the typical case shown in Figure 2. However, BUS and rBUS yielded similar *sr*s and time-efficiencies in the experiments, which reveals the sign of bias angle do not account for the low *sr*s and time-efficiencies of BUS. A possible reason is that BUS succeeded with the small rotating angle, *i.e.*, 10°, in biasing the robot heading towards the departing plume for more times in M group than in S and N groups. In real applications, the fluctuation of airflow field is unpredictable. Optimizing the bias angle beforehand to adapt various unknown airflow fields is infeasible in real-world CPT missions.

cVTF yielded similarly high *sr*s, *i.e.*, 58.57% (M group), 48.91% (S group), and 50.13% (N group), and time-efficiencies in the three groups. Even without collaboration, VTF produces higher *sr*s and time-efficiencies than BUS and rBUS in S and N groups. This reveals that learning the bias angle for Track-Out activities in an online manner is a feasible solution to rapidly re-contacting the lost plume in real CPT problems. Since the bias angle is learned at the beginning of individual Track-Out activities, the robots can learn from recently updated VTs, which enable the adaptation to different environments. Moreover, cVTF yielded higher *sr*s and time-efficiencies than VTF. Due to the ε-greedy selection mechanism used in the process of policy improvement, only good VTs with high Q-value are stored in the VT database. The shared VT database maintained by multiple robots usually contains much more good VTs than VT databases that are maintained independently. Thus, the probability of yielding rapid and successful Track-Out activities is higher for cVTF than for VTF. In addition, VTF and cVTF provide an invariant robot heading during each individual Track-Out activity, while BUS produces new robot heading for the robot in each cycle. Thus, VTF and cVTF reduce the communication burden needed for controlling the robots. Although VTF and cVTF yielded higher *rdo*s and lower efficiency with respect to the robot trajectory than BUS and rBUS, such a drawback can be compensated by their higher *sr*s. Failed Track-Out activities will trigger the plume reacquiring behavior, which usually outputs much more winding routes (e.g., clover-leaf-shaped routes [10]) for the robot and deteriorates the overall distance overhead of the whole CPT method.

Two major limitations of the experimental results are as follows: First, the necessity of optimizing the bias angle is based on the assumption that the robot requires longer time to rotate over a bigger angle. If the robot can realize an ideal rotating, *i.e.*, rotating over any angle can be accomplished immediately, utilizing a new robot heading perpendicular to the current wind direction along the right direction might be optimal in most cases. Nevertheless, in the case of ideal rotating, VTF can be modified for learning the optimal sign of bias angle. Second, only three groups of experiments were conducted in normal airflow fields. The performance of our methods are not tested in more complicated environments, e.g., turbulent environments. Thus, we declare the applicability of our methods within a limited range.

## 6. Conclusions

We have proposed an instance-based RL method and its collaborative version, namely VTF and cVTF, for learning the bias angle used in Track-Out activity to rapidly re-contact the lost plume during the process of CPT. The Track-Out activity, which biases the robot heading relative to upwind direction, is activated in the time immediately following the loss of chemical detection. In VTF, the robots learn from their recently stored instances of successful Track-Out activities. Through collaboration, the robots learn from their own instances and the instances shared by other robots in cVTF.

With respect to our experimental conditions, VTF and cVTF yielded generally higher success rates and time-efficiencies than BUS. VTF and cVTF realize online learning based on recently stored instances of successful Track-Out activities. In contrast, BUS utilizes an offline optimized bias angle through all Track-Out activities. Therefore, VTF and cVTF can adapt to different environments, while it is hard to optimize the bias angle beforehand for BUS with respect to all possible environments. Moreover, cVTF yielded higher success rates and time-efficiencies than VTF. Since there are more instances of rapidly succeeded Track-Out activities shared in cVTF than those maintained independently in VTF, cVTF yields higher probability of rapidly re-contacting the plume than VTF.

**Figure 16:**
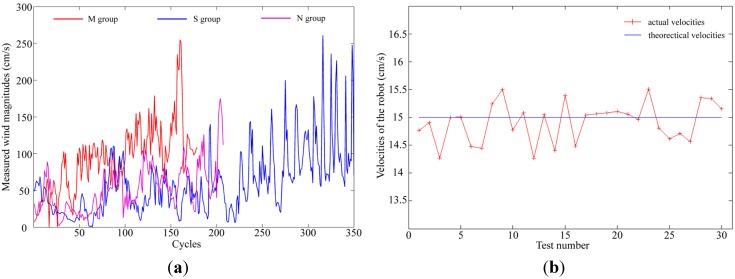
(**a**) Typical wind magnitudes measured in the three groups of experiments; (**b**) The robot’s actual velocities recorded in 30 tests.

As mentioned in Section 4.1, the robots’ theoretical velocity vectors were subtracted from the measured relative wind velocity vectors to calculate the absolute wind velocity vectors. Through this calculation, noises can be introduced by the robot’s movements into the absolute wind velocities. Nevertheless, it is feasible to neglect the introduced noises, since they are rather minor compared with the measured relative wind velocities. Typical wind magnitudes measured in the three groups of experiments are shown in Figure 16a. Errors introduced by the robot’s movements were assessed by averaging the differences between the theoretical and actual velocities of the robot in 30 tests, which are shown in Figure 16b. In each test, the robot was manuvered for 3 meters with a theoretical forward velocity of 15 cm/s. The actual velocity was recorded as the result of dividing 3 meters by the actual spent time. While the majority of measured wind magnitudes ranged from 30 cm/s to 200 cm/s, the robot’s movements only introduced small errors of less than 1 cm/s.

## Appendix

Table A1 lists the notation used in this paper.

**Table A1:** The notation used in this paper.

$T_{L}$	Number of cycles from the last chemical detection event till the current time.
λ	Cycle limit for the Track-Out activity.
$T_{Re}$	Cycle limit for the plume re-acquiring behavior.
θ(k);θ	Robot heading at the *k*-th cycle for BUS; Robot heading learned by VTF/cVTF.
β(k);β	Bias angle at the *k*-th cycle for BUS; Bias angle learned by VTF/cVTF.
$\psi (k),\psi_{L}$	Angle of wind direction measured at the *k*-th cycle and LDP, respectively.
$x(k),x_{L}$	Position of the robot at the *k*-th cycle and LDP, respectively.
$s_{k},a_{k},r_{k}$	State, action, and reward at the *k*-th cycle, respectively.
$Q^{\pi }(s,a)$	Action value when action a is conducted at state s and thereby following policy π.
α	Learning rate used in VTF/cVTF.
γ	Discount rate used in VTF/cVTF.
ε	Probability of selecting random action in the ε-greedy selection mechanism.
M	Number of actions.
$u.s,u.s^{\prime },u.Q$	Start state, end state, and Q-value of the new VT u, respectively.
$\tilde{u}$	Nearby VT of u.
${\hat{u}}_{a}$	The VTs associated with action a.
U	Database for storing VTs.
N	Size limit of .Size limit of U.
$w_{ij}$	The weight for the *j*-th VT associated with action $a_{i}$.
c(k)	Transient concentration measurement at the *k*-th cycle.
$\bar{c}(k)$	Adaptive concentration threshold at the *k*-th cycle.
δ	Constant parameter for calculating $\bar{c}(k)$.
$x_{g}=\{x_{g},y_{g}\}$	Goal position of the robot.
$d_{th}$	Distance threshold for determining whether to generate repulsive force or not.
$d_{big}$	A distance that is big enough for APF to generate sufficient attractive force.
$n_{t}$	Number of times that the robot has arrived at $x_{g}$ in “casting” behavior.
$d_{ss}$	Scanning span added to the scanning width in “casting” behavior.
$v_{max}$	The maximal velocity of the robot.
